# Emphysematous cystitis in an elderly Japanese patient with type 2 diabetes mellitus

**DOI:** 10.1002/ccr3.2028

**Published:** 2019-02-06

**Authors:** Hironori Yashima, Michishige Terasaki, Hideki Kushima, Tsutomu Hirano

**Affiliations:** ^1^ Department of Medicine, Division of Diabetes, Metabolism and Endocrinology Showa University School of Medicine Tokyo Japan

**Keywords:** diabetes mellitus, emphysematous cystitis, *Escherichia coli*, high mortality rate

## Abstract

We report a rare but fatal case of emphysematous cystitis (EC) that presented as a typical image of gas bubbles within the urinary bladder wall on computed tomography (CT). This disease has a high mortality rate more than that reported previously based on recent literature review in Japan.

## 1 CASE REPORT

**Figure 1:**
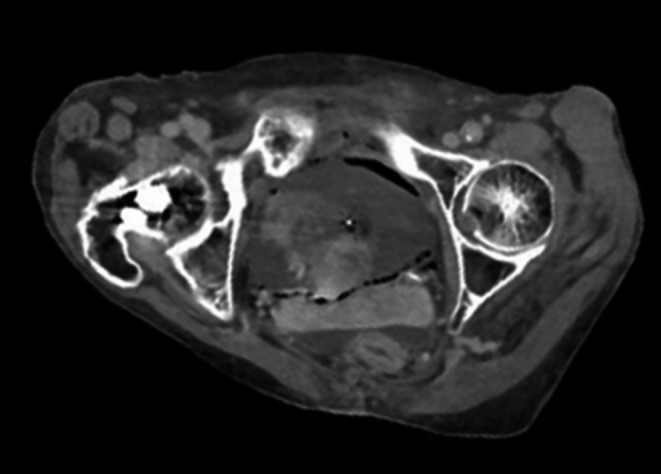
Computed tomography of the abdomen and pelvis shows diffuse gas collection within the urinary bladder wall (Transverse plane)

**Figure 2:**
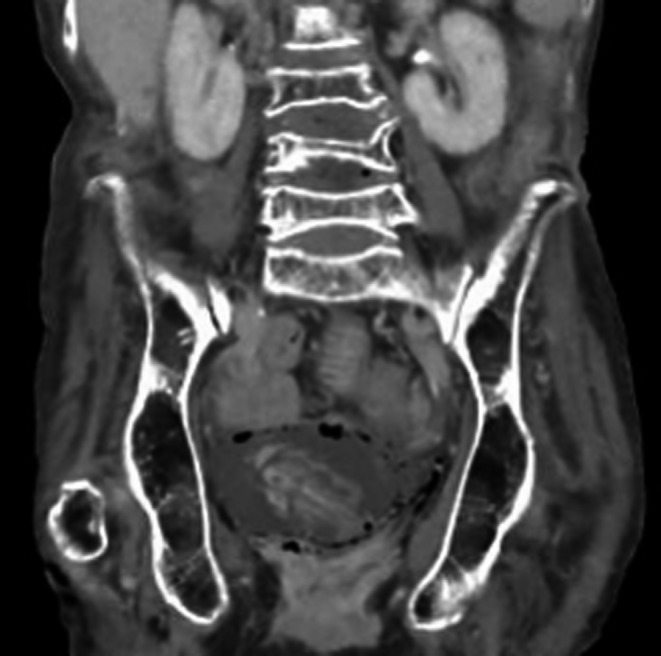
Computed tomography of the abdomen and pelvis shows diffuse gas collection within the urinary bladder wall (Coronal plane)

**Figure 3:**
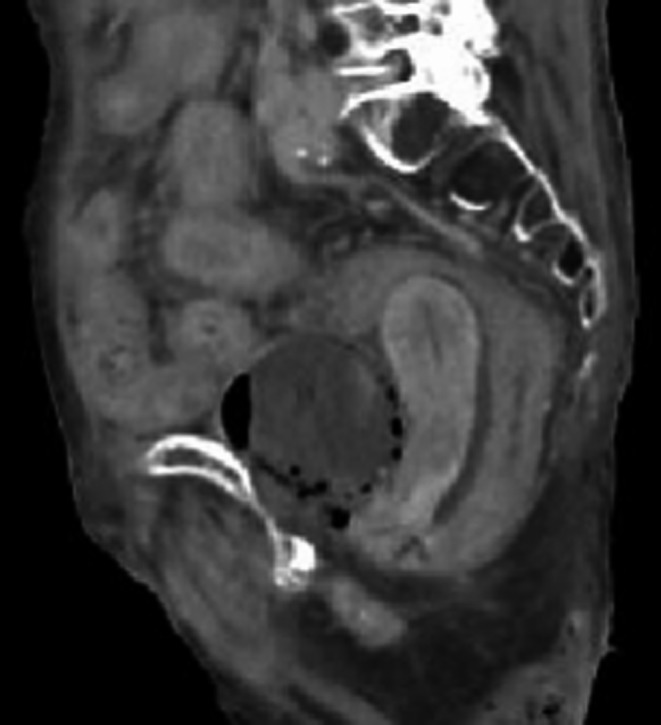
Computed tomography of the abdomen and pelvis shows diffuse gas collection within the urinary bladder wall (Sagittal plane)

An 85‐year‐old woman with type 2 diabetes mellitus presented to the emergency department of our hospital with coma and shock. She had not visited any hospital previously, had never been examined for complications of diabetes, and had never consumed any medications before. On admission, her vital signs were as follows: consciousness, Glasgow Coma Scale score 3/15 (E1V1M1); blood pressure, 80/40 mm Hg; pulse rate, 130 bpm; and temperature, 37.3°C. Laboratory analysis revealed neutropenia (neutrophils count, 640/µL) and elevated C‐reactive protein level to 12.73 mg/dL. The levels of blood glucose and hemoglobin (Hb)A1c were 237 mg/dL and 12.1%, respectively. Blood and urine cultures showed the presence of *Escherichia coli*. CT of the abdomen and pelvis without contrast revealed diffuse gas collection within the urinary bladder wall (Figures 1, 2, 3), which is a typical sign of EC caused by gas‐forming bacteria.^1^, ^2^ The patient was intensively treated with a broad‐spectrum antibiotic meropenem hydrate and a vasopressor, and was put on respirator and catheterized; however, she died within 2 days due to circulatory failure. Based on literature review, including our study, from 2016 to 2018 in Japan, the mortality rate of EC is 26% (Table S1). This study may provide a new insight into this disease.

## CONFLICT OF INTEREST

None declared.

## AUTHOR CONTRIBUTION

HY: treated the patient and researched the literature. MT: drafted, revised, and finalized the manuscript. HK: treated the patient. TH: reviewed the manuscript. All authors approved the final version of the manuscript.
